# Mechanobiology of Adipocytes

**DOI:** 10.3390/biology13060434

**Published:** 2024-06-13

**Authors:** Sean P. Blade, Dylan J. Falkowski, Sarah N. Bachand, Steven J. Pagano, LiKang Chin

**Affiliations:** 1 Department of Biomedical Engineering, Widener University, Chester, PA 19013, USA; spblade@widener.edu (S.P.B.); djfalkowski@widener.edu (D.J.F.); snbachand@widener.edu (S.N.B.); 2 Department of Mechanical Engineering, Widener University, Chester, PA 19013, USA; sjpagano@widener.edu

**Keywords:** mechanobiology, adipocyte biology, obesity, finite element analysis

## Abstract

**Simple Summary:**

The rising population of people with obesity has presented an important need for the study of how adipocytes (fat cells) behave and affect biological function. Adipocytes have traditionally been thought to only function as energy storage for the body, an assumption that has led to them being understudied in health sciences. However, recent studies have shown that adipocytes experience forces in vivo, are reactive to mechanical stimuli, and their cell function can be altered by changes to the mechanical microenvironment and, in some cases, contribute to disease. The aim of this review is to summarize recent scientific publications on how adipocytes sense and respond to their mechanical environment, the use of engineered scaffolds to study cell behavior, adipose cell and tissue mechanical properties, and the current state of computational models.

**Abstract:**

The growing obesity epidemic necessitates increased research on adipocyte and adipose tissue function and disease mechanisms that progress obesity. Historically, adipocytes were viewed simply as storage for excess energy. However, recent studies have demonstrated that adipocytes play a critical role in whole-body homeostasis, are involved in cell communication, experience forces in vivo, and respond to mechanical stimuli. Changes to the adipocyte mechanical microenvironment can affect function and, in some cases, contribute to disease. The aim of this review is to summarize the current literature on the mechanobiology of adipocytes. We reviewed over 100 papers on how mechanical stress is sensed by the adipocyte, the effects on cell behavior, and the use of cell culture scaffolds, particularly those with tunable stiffness, to study adipocyte behavior, adipose cell and tissue mechanical properties, and computational models. From our review, we conclude that adipocytes are responsive to mechanical stimuli, cell function and adipogenesis can be dictated by the mechanical environment, the measurement of mechanical properties is highly dependent on testing methods, and current modeling practices use many different approaches to recapitulate the complex behavior of adipocytes and adipose tissue. This review is intended to aid future studies by summarizing the current literature on adipocyte mechanobiology.

## 1. Mechanobiology

Mechanical stimuli affect cell function and numerous biological processes including development [1], homeostasis [2], tissue repair [3], and disease progression [4,5,6]. The study of how physical signals are sensed, transduced, and how they regulate cell behavior is termed mechanobiology and is a growing area of research that integrates concepts across many disciplines, including structural engineering, biomechanics, biophysics, and cell and molecular biology. A pioneer of the field is Julius Wolff, M.D. (1836–1902), a German surgeon who observed that trabecular bone remodels along new lines of tensile and compressive forces after fracture [7]. Since then, our understanding of mechanical signaling and its consequences has grown substantially, largely in cells and tissues of the musculoskeletal system which function to bear load, but knowledge of mechanobiology in non-weight bearing soft tissues continues to grow.

### 1.1. Transmission of Mechanical Loads to the Nucleus

All tissues are constantly under static and dynamic mechanical loads. Examples include flow-induced shear stress through the circulatory and lymphatic systems as well as compressive forces on cartilage and bone. Any pushing, pulling, or twisting of the extracellular matrix (ECM) is felt by resident cells through two notable mechanisms. First, the ECM has a direct physical connection to the nucleus of the cell, starting with transmembrane receptors such as integrins and cadherins that bind to ECM ligands like collagen, fibronectin, elastin, and laminin [8]. Receptor–matrix binding initiates the formation of focal adhesions, highly dynamic macromolecular structures most notably consisting of the proteins talin, paxillin, and vinculin that serve as bridges between the ECM and the intracellular actin cytoskeleton [9]. Any reorganization of the actin network can, in turn, be transmitted to the nucleus through the linker of nucleoskeleton and cytoskeleton (LINC) complex, which primarily consists of the Sad1p and Unc-84 (SUN) proteins, and the Klarsicht, ANC-1, and Syne homology (KASH) proteins [10]. Through this physical connection, any mechanical perturbation of the ECM can be directly transmitted to the nucleus, consequently affecting cell behavior and function. Second, mechanical inputs to the cell are transduced into biochemical or electrical signals, a process termed mechanotransduction [11]. Through cell membrane-associated mechanosensitive proteins including but not limited to ion channels, G-protein-coupled receptors and kinases as well as integrins, the cell is able to perceive mechanical stimuli, triggering mechanosignaling cascades [12]. Several well-established mechanotransduction pathways are Wnt [13], Notch [14], RhoA/Rho-kinase (ROCK) [15], and Hippo [13]. Ultimately, these mechanical cues can influence cell function and behavior including morphology [16], motility [16], proliferation [16], metabolism [17,18], and differentiation [15,19], as well as tissue-level characteristics such as organization and function [20].

### 1.2. Lamins

Mechanical stimuli propagated via the actin network and the LINC complex to the nucleus meet the meshwork of nuclear structural proteins called lamins. Recent advances in the study of mechanobiology have led researchers to uncover the role of nuclear laminar structural proteins in maintaining nuclear mechanical properties and shape, cell differentiation, and cell functions. These intermediate filament proteins (type V) primarily exist in the inner nuclear membrane as a meshwork interface between chromatin and the nuclear membrane [21,22,23,24,25]. The four major isoforms of lamins (A, B1, B2, and C) have unique impacts on the differentiation of cells. The B-type lamins are present in cells, regardless of their differentiation state and are thought to play a role in cell proliferation, differentiation, and gene expression. Cells with B-type lamins silenced will apoptose, and overexpression of B type leads to increased proliferation and life span. A and C lamin, however, are only present during differentiation [21]. Lamin A/C-deficient cells exhibit altered mechanical properties, becoming more susceptible to strain and therefore have decreased nuclear stiffness [26]. Knockdown of an individual lamin will result in abnormally shaped nuclei [27], thus confirming the role of lamin proteins as a major structural element of the nucleus, similar to how cytoskeletal IFs mediate the shape and mechanical properties of the cell [24,25]. Since irregularly shaped nuclei are known to alter gene expression, disruption to lamin proteins by mechanical events can significantly change cell function [28]. There is still much to be uncovered regarding the role of lamins, especially in adipocytes.

### 1.3. Significance of Mechanobiology in Normal Function and Disease

Cellular mechanobiology has manifestations seen on the tissue/organ-level through the development of tissues and organs as well as through disease. One example of tissue-level mechanobiology is heart development. Cardiac myocytes are under constant mechanical stress as a consequence of self-induced beating and hemodynamics, which can drive the differentiation of stem cells during development [29,30]. Congenital heart disease can occur if these mechanical cues are absent or abnormal. Another example is cancer cell migration. One of the driving forces that transforms healthy cells into cancerous cells is disruptions of the ECM and substrate mechanics, including cell–matrix interactions [31,32]. On the opposite side of the same coin, the therapeutic effects of using mechanosensitive proteins as potential targets and/or biomarkers of cancer have been studied. The widely used cancer drug taxol acts not only by inhibiting mitosis through microtubule stabilization, but also by disrupting cellular mechanosensing and the transition of the cell into an invasive phenotype [33]. Strategies to deplete cancer-associated fibroblasts and decrease ECM deposition, thereby decreasing solid stress in tumors, also show promise as cancer treatments [34]. These examples demonstrate the impact of mechanobiological processes on physiological systems and function.

## 2. Adipocyte Mechanobiology

While the mechanobiology of cells in load-bearing tissues, such as osteoblasts, chondrocytes, and tenocytes, are well-studied, less is known about the mechanobiology of adipocytes. Once thought of as inert cells that function only to store excess energy, adipocytes are now recognized as extremely active cells that are involved in cellular communication [35,36], inflammation by secreting mediators known as adipokines [37,38], metabolism [38], and systemic homeostasis [38]. A growing body of knowledge confirms that adipocytes are indeed mechanosensitive [17,39].

### 2.1. Diverse Roles of Adipocytes

Adipocytes fulfill diverse roles and are distributed throughout different regions of the body. Adipocytes maturing to form white adipose tissue (WAT) are responsible for energy storage [40]. WAT specializes in sequestering free fatty acids away from vital organs such as the liver, heart, and muscles. Additionally, white adipocytes are highly involved in cellular communication with numerous other cell types through the secretion of exosomes, thus regulating whole body metabolism. Moreover, adipokines and cytokines released by white adipocytes function to link metabolism with the innate and adaptive immune systems. Those adipocytes that mature to form brown adipose tissue (BAT) facilitate thermoregulation and insulation by dissipating energy in the form of heat [41]. Despite its physiological significance, BAT is not involved in obesity like WAT.

Adipose tissue is distributed throughout various regions of the body; the two main depots being subcutaneous and visceral fat [42,43]. Subcutaneous adipose is positioned directly below the skin, providing insulation, while visceral fat is located within the abdomen, acting as a protective cushion for internal organs. More importantly, in the context of obesity, these two fat depots exhibit distinct metabolic activity. Subcutaneous fat primarily functions in thermogenesis, whereas visceral fat is highly metabolically active and releases cytokines associated with inflammation. Abnormal increases in the relative amount of visceral fat compared to total fat are linked to disorders of lipid and glucose metabolism.

### 2.2. Cellular Structure

**Figure 1:**
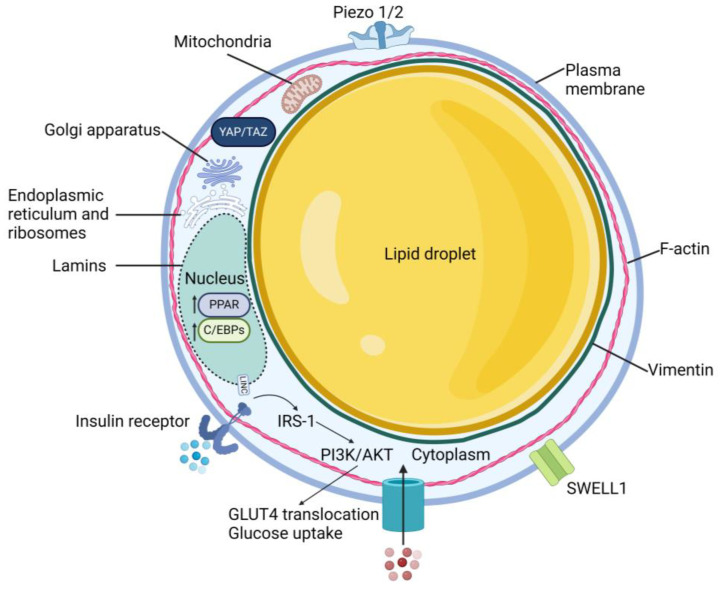
Schematic of the adipocyte and relevant mechanosensitive proteins, including Piezo 1/2 (2.4 Piezo channels), F-actin (2.2 Cellular structure and 4. Cell stiffness measurements), vimentin (2.2. Cellular structure), SWELL1 (2.5. SWELL1), lamins (1.2. Lamins and 2.2. Cellular structure), PPAR (3.2. Response to shockwaves and 3.4. Response to underlying substrate stiffness), C/EBPs (3.2. Response to shockwaves and 3.4. Response to underlying substrate stiffness), IRS-1 (3.4. Response to underlying substrate stiffness), PI3K/AKT (2.5. SWELL1), and GLUT4 (3.4.1. Effects on insulin sensitivity). Not drawn to scale. Created with BioRender.com (accessed on 10 March 2024).

**Figure 2:**
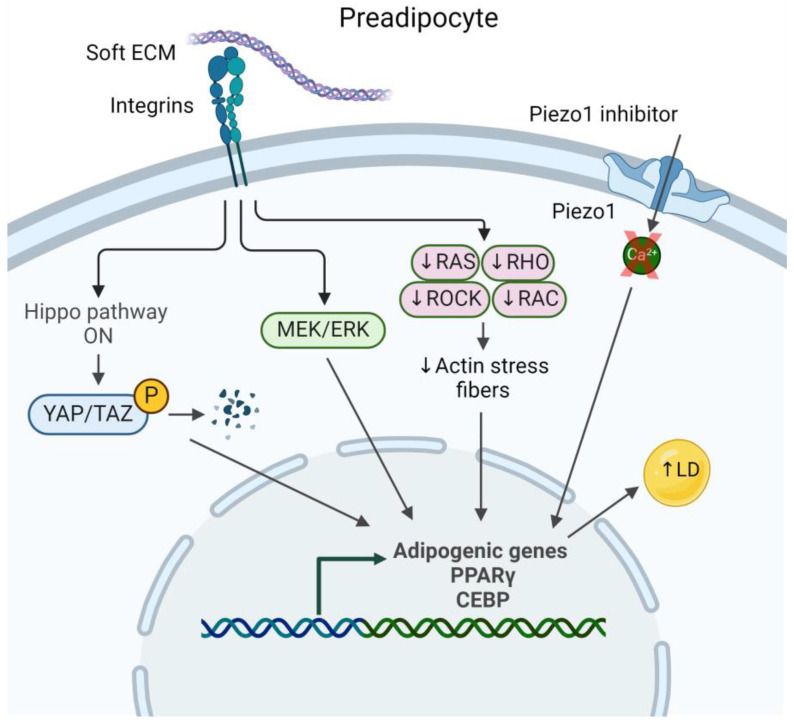
Schematic of the preadipocyte and relevant mechanotransduction pathways and mechanosensitive proteins involved in differentiation. ECM = extracellular matrix, LD = lipid droplet, P = phosphorylation. Not drawn to scale. Created with BioRender.com (accessed on 7 June 2024).

Mature adipocytes possess a unique cellular structure, consisting of one or a few large lipid droplets surrounded by a thin layer of cytoplasm and a plasma membrane (Figure 1). Within the cytoplasmic compartment are several organelles including the nucleus, Golgi apparatus, endoplasmic reticulum, ribosomes, and mitochondria [44]. Actin stress fibers are lacking; instead, F-actin forms a cortical actin network close to the cell membrane [45,46,47]. As preadipocytes differentiate, they downregulate Ras homolog family member A (RhoA) to destabilize the actin cytoskeleton to allow the maturing adipocyte to roundup thus accommodating the growing lipid droplet, which then pushes actin against the cell membrane (Figure 2). In the absence of prominent actin stress fibers and a robust cytoskeletal network, how is force physically transmitted from the ECM to the nucleus? Perhaps the lipid droplet plays a role, as well as the intermediate filament vimentin, which surrounds the nucleus and extends throughout the cytosol in preadipocytes, but upregulates and reorganizes to form a cage around the lipid droplet during adipocyte differentiation [48]. Yet, this hypothesis needs to be further explored. The nuclear lamina also rearranges during differentiation; adipocytes express greater amounts of lamins A, C, and B1 at the nuclear rim, but decreased total lamin A/C with maturation [49,50]. The reorganization of nuclear lamins during adipogenesis could lead to softening of the nuclear membrane, enabling the expanding lipid droplet, typically softer than the nucleus under normal conditions, to deform the nucleus.

### 2.3. Yes-Associated Protein

Several mechanoregulatory proteins have roles in adipocyte differentiation and function. Yes-associated protein (YAP) and the transcriptional coactivator with PDZ-binding motif (TAZ) are transcriptional cofactors of the Hippo pathway that bind to the transcriptional enhanced associate domain (TEAD) family of transcriptional factors and are key regulators in the differentiation of mesenchymal stem cells (MSCs) [51,52,53,54]. The phosphorylation of YAP leads to its retention in the cytoplasm and its subsequent degradation, and these events are associated with an adipogenic phenotype (Figure 2). Localization of YAP/TAZ can be controlled by mechanical inputs, such as substrate stiffness [55,56], shear stress as a result of fluid flow [57], or stretching [58]. Activation of YAP/TAZ influences cell growth, development, and proliferation [53].

### 2.4. Piezo Channels

**Figure 3:**
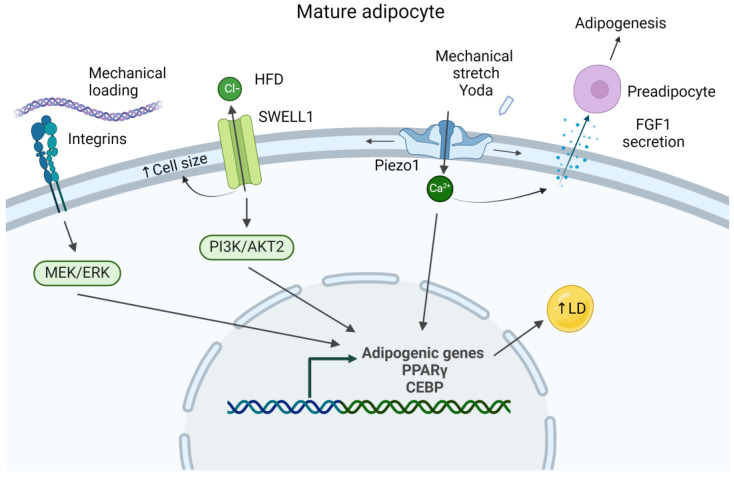
Schematic of the mature adipocyte and relevant mechanotransduction pathways and mechanosensitive proteins involved in mediating adipogenesis, cell size, and differentiation. HFD = high-fat diet, LD = lipid droplet. Not drawn to scale. Created with BioRender.com (accessed on 7 June 2024).

Several ion channels play crucial roles in adipocyte function, such as voltage-gated calcium channels, transient receptor potential channels, and store-operated calcium channels [59]. However, this review will concentrate on two specific ion channels recognized as mechanosensitive proteins—Piezo1/2 and SWELL1. Found in subcutaneous, visceral, and perivascular adipose tissue [60], Piezo channels, when activated, promote the conversion of adipocytes to pro-fibrotic fibroblasts (Figure 2). Recently, Griffin et al., demonstrated that when adipocytes seeded into a 3D hydrogel were stretched (the amount and duration of strain were not reported), they obtained a fibroblast-like phenotype with decreased expression of adiponectin but increased expression of collagen and alpha-smooth muscle actin [61]. The effect of mechanical stretching was eliminated when small molecules were used to inhibit Piezo1, Piezo2, YAP, or focal adhesion kinase (FAK). Adipocytes collected from human liposuction procedures that were treated with Yoda1, an agonist of Piezo1, exhibited hypertrophy with or without stretching (Figure 3). Conversely, inhibition of Piezo1 or Piezo2 yielded a greater number of adipocytes and fewer adipocyte-derived fibroblasts compared to unstretched controls as evidenced by increased adiponectin levels and less collagen 1 staining, respectively. Similar results were obtained in an in vivo mouse model, where stretching (again, amount and duration of strain were not reported) was applied to subcutaneous wounds. Scars appeared to be more fibrotic, have increased adipose-derived fibroblasts, and increased Piezo1 and Piezo2, as shown by immunohistochemistry. Recently, Wang et al., demonstrated that Piezo1 stimulates the mature adipocyte to release adipogenic fibroblast growth factor 1, which mediates the adipogenic differentiation of neighboring precursor cells (Figure 3) [62].

### 2.5. SWELL1

SWELL1 channels have also been identified as part of the adipocyte mechanosensing machinery. SWELL1 is a volume-regulated anion channel that regulates adipocyte size, thus allowing for cell expansion [63]. Additionally, it is required for insulin–phosphoinositide 3-kinases(PI3K)-AKT2 signaling and therefore controls insulin sensitivity and glucose uptake. When fed a high-fat diet, mice with an adipose-specific SWELL1 knockout exhibited a 20% decrease in adipose tissue mass due to a decrease in adipocyte cell size and disrupted insulin/PI3K/AKT2 signaling (Figure 3) [64]. Interestingly, male adipose-specific SWELL1 knockout mice fed a high-fat high-sugar diet exhibited a 43% increase in hepatic steatosis compared to wild-type mice due to diminished triglyceride storage, underscoring the potentially detrimental systemic effects that can stem from adipocyte dysfunction.

## 3. Mechanical Response of Adipocytes

Adipogenesis is a cellular process that contributes to the expansion of adipose tissue in obesity. Obesity is a medical condition in which there is an accumulation of white adipose tissue (WAT) as a result of the hypertrophy and hyperplasia of adipocytes [65]. Mouse models show that both adipocyte size and number increase in obese adipose tissue, while human models also show that preadipocytes conserve the ability to differentiate into adipocytes and therefore continually contribute to adipose growth. Adipocyte differentiation is a multi-step, dynamic morphological process controlled by transcriptional factors, which in addition to the accumulation of lipid droplets, gives rise to the final phenotype. Adipogenesis is now recognized as a mechanosensitive process; there is strong evidence to show that the formation of the lipid droplet depends on the mechanical environment of the cell.

Although experimental methods, strain amplitude, and duration vary between studies, several research groups have shown that cyclic stretching [66,67,68,69,70], cyclic compression [71,72], and vibration [73] suppress adipocyte differentiation, as shown by a downregulation of the master regulator proliferator-activated receptor γ (PPARγ). In contrast, static stretch, which mimics the loading experienced during a sedentary lifestyle linked to obesity, tends to induce adipocyte differentiation [74]. Herein, we discuss two additional studies published in the past several years that add to this body of knowledge.

### 3.1. Response to Tensile Strain

A recent study by Moldovan et al., investigated changes in the mass spectrometry profile of 3T3-L1 cells differentiated on flexible substrates with or without applied stretch [75]. Cells that were subjected to a constant 12% tensile strain for 19 days highly express several mitochondrial proteins such as NADH:ubiquinone oxidoreductase subunit A10 (Ndufa10), Ndufb8, Ndufv1, ATPase H+ transporting V1 subunit e1 (Atp6v1e1), and Atp5f1 as well as an increase in mitochondria compared to unloaded controls. Lipid formation requires acetyl coenzyme A and reduced nicotinamide adenine dinucleotide (NADH), both of which are byproducts of mitochondrial activity, thus indicating that adipocytes experienced a higher rate of metabolism under the static stretch condition. Morphologically, adipocytes under stretch conditions had fewer lipid droplets, but the average lipid droplet radius was 30% greater compared to controls. Findings of this study support the current understanding that static stretch accelerates adipogenesis and adipocyte maturation [74]. The consequences of static stretch may, therefore, reflect the physiological conditions in obesity that lead to the hyperplasia and hypertrophy present in obese adipose tissue.

### 3.2. Response to Shockwaves

The effects of low-energy shockwaves on adipocyte differentiation have also been studied [65]. Shockwave therapy is commonly used to treat kidney stones, but has proven therapeutic benefits in musculoskeletal tissue repair likely by promoting neovascularization and angiogenesis as well as reducing matrix metalloproteinases, amongst other actions. When either 3T3-L1 preadipocytes or human subcutaneous preadipocytes were given shockwave treatment (0.025 mJ/mm^2^, 10 Hz, 1000 impulses) throughout a standard differentiation protocol, adipocyte differentiation was inhibited. While the number of adipocytes were no different between shockwave-treated cells and controls, both mouse and human adipocytes had 15% less intracellular lipid droplets with treatment as visualized by Oil red O staining. Moreover, treated adipocytes had decreased expression of various adipocyte markers, quantified by a quantitative reverse transcription–polymerase chain reaction. These markers include transcription factors PPARγ1/2 and CCAAT/enhancer-binding-protein α (CEBP), lipid droplet-associated proteins perilipin1 and fat-specific protein 27 (Fsp27), and the lipid synthesis proteins fatty acid synthase (FAS) and acetyl-coA carboxylase (ACC). Western blot analysis confirmed that the protein amount also decreased for the aforementioned adipocyte markers, but not for Fsp27 which was not quantified. To tease out possible mechanisms, further immunoblotting experiments were performed. They showed that the application of shockwaves induced the release of extracellular adenosine triphosphate (ATP), previously found to be involved in adipocyte differentiation, and decreased intracellular cyclic adenosine monophosphate (cAMP), a second messenger essential for PPARγ activation. Furthermore, the addition of endogenous cAMP to shockwave-treated cells was able to recover PPARγ protein levels, possibly through the Wnt/β-catenin pathway. Interestingly, the application of shockwaves had no effect on PPARγ for fully differentiated mature adipocytes, thus highlighting the role of PPARγ in adipocyte differentiation, but not mechanosensing or mechanotransduction.

### 3.3. Response to Compression

One important consideration to mention is that most studies that investigate adipocyte mechanobiology focus on the role of mechanical stimuli in the differentiation of preadipocytes; in other words, very few studies investigate the mechanoresponsiveness of mature adipocytes. Although it is certainly of great interest to explore how loading regimes attenuate adipogenesis and therefore prevent WAT expansion, it is equally important to understand how mechanics affect the function of the mature adipocyte. Additionally, identification of the associated mechanosensitive pathways can help identify potential therapeutic targets. We are only aware of one study in the past several years that evaluates the effect of mechanical loading on mature adipocytes from WAT. Pellegrinelli et al., embedded mature adipocytes isolated from the subcutaneous WAT of lean human subjects into three-dimensional (3D) peptide hydrogels with or without decellularized tissue digest from obese human subjects (dMAT) [76]. Static compressive strains of 0–50% applied by placing small columns on top of the 3D constructs sans dMAT resulted in a dose-dependent decrease in glycerol release into the medium and an increase in granulocyte-colony stimulating factor (G-CSF), interleukin-8 (IL-8), and interleukin-6 (IL-6), similar to the response seen when adipocytes were embedded in 3D hydrogels with dMAT but no load. These results suggest that static compression induces a pro-inflammatory response similar to that observed in obesity.

### 3.4. Response to Underlying Substrate Stiffness

Adipocytes live in a sparse ECM made of collagen, fibronectin, and laminin [77]. How changes to the ECM—whether it be alterations in substrate elasticity, composition, or applied forces—exacerbate or promote adipose tissue dysfunction is still unknown. What is established is that the maladaptive ECM remodeling that takes place because of obesity contributes to systemic dysfunction, disrupting the performance of skeletal muscle, the heart, liver, and pancreas [78]. These tissues are central for controlling metabolism. Obesity increases the risk for chronic illness such as insulin resistance, diabetes, heart disease, nonalcoholic fatty liver disease and nonalcoholic steatohepatitis [64], atherosclerosis [78], and hypertension [79].

ECM protein composition and their interactions, along with other molecules such as adipokines [80], change during diseases such as obesity, and these changes are typically accompanied by altered tissue stiffness. Additionally, the ECM cross-linking enzyme lysyl oxidase [81] increases with fibrosis, contributing to stiffer tissues. All of these changes to the ECM have been shown to affect normal cell function [17,19,82].

#### 3.4.1. Effects on Differentiation

It is well established that MSCs cultured on soft substrates tend to differentiate into adipocytes and those cultured on stiff substrates are steered towards an osteoblastic lineage [19]. When cultured on substrates that mimic wild-type adipose tissue, cells adopt and maintain a more contracted shape and smaller aspect ratio than cells on stiff substrates [83]. This morphological change precedes any lipid accumulation that differentiating MSCs might exhibit. In addition to morphology, adipogenic differentiation was confirmed by the gene expression of adipogenic markers such as PPARγ, C/EBPs, and fatty acid-binding protein 4 (FABP4). Interestingly, when actin polymerization is inhibited by the addition of cytochalasin-D, thus lowering cytoskeletal tension [30], MSCs display a round morphology and lipid vacuoles regardless of underlying substrate stiffness [83]. In contrast, cell spreading and stiff substrates are associated with osteoblast differentiation. Although extensively used to demonstrate how MSC differentiation can be controlled by mechanics, it is important to note that the use of cell scaffolds with tunable stiffness and/or ECM ligand concentration as tools to study adipocyte mechanobiology is limited.

More recently, Takata et al., investigated the effects of substrate stiffness on beige adipocyte differentiation and function. The beige adipocyte is present in inguinal WAT (iWAT) and is characterized by high levels of uncoupling protein 1 (UCP1), a mitochondrial protein involved in the production of heat [18]. A rigid substrate promotes iWAT cell spreading with the cell area being 2.1-fold larger on stiff substrates compared to the soft ones. Additionally, stiff substrates promoted the differentiation of iWAT cells to beige adipocytes. Several key genes of adipocyte markers—uncoupling protein 1 (UCP1) and PR domain-containing 16 (PRDM16)—were expressed more by cells on stiff substrates after isoproterenol induction compared to cells on soft substrates. Markers for white adipocytes, such as resistin and adipocyte protein 2 (aP2), were no different for cells seeded onto soft and stiff substrates, indicating that a rigid substrate in this experimental setting promoted differentiation into beige-type adipocytes, but not white adipocytes.

#### 3.4.2. Effects on Insulin Sensitivity

Substrate stiffness has been shown to directly affect insulin sensitivity. Li et al., demonstrated that when differentiated 3T3-L1 adipocytes were seeded onto collagen- and fibronectin-coated polyacrylamide gels with stiffness equivalent to adipose tissue (250 Pa) and treated with insulin, glucose transporter 4 (GLUT4) recruitment to and insertion into the cell membrane increased compared to cells seeded onto stiffer gels or glass [17]. GLUT4 is a glucose transporter that translocates from its intracellular locations to the plasma membrane under the regulation of insulin. Additionally, adipocytes demonstrated increased expression of insulin receptor substrate-1 (IRS-1) and AKT, key regulators in glucose and lipid metabolism. Although not particularly recent, these results are noteworthy as they suggest that insulin sensitivity is optimized when adipocytes are on an ECM of normal elasticity and deviations in substrate stiffness may be connected to insulin resistance.

## 4. Cell Stiffness Measurements

Atomic force microscopy (AFM) was originally developed as a tool to procure nano-scale topological information and characterize surface roughness [84]. A physical cantilever tip taps, scans, or indents the surface of a sample, while the reflection of a laser focused on the back of the tip is captured by a photodiode. The known material properties of the cantilever and its deflection can be used to determine structural and material properties. The driving principle behind this technique is the assumption that the AFM cantilever and the sample act as springs in series. A known force or displacement can be applied, and the measured deflection of the cantilever is representative of the response of the sample [85,86]. AFM is a breakthrough technique for evaluating cell stiffness and is used extensively to measure the biomechanical properties of the nucleus, cytoplasm, and other organelles in many different cell types [87,88,89]. However, since adipocytes have only recently been thought of as mechanosensitive cells, only a handful of published studies characterize adipocyte stiffness using AFM [87,90,91,92].

Shoham et al., used AFM to determine the effective stiffness (ES) of adipocytes at locations containing lipid droplets or the nucleus over the course of 11 to 19 days after induction of adipogenic differentiation of 3T3-L1 preadipocytes [90]. It was determined that adipocytes stiffen with the time postinduction of differentiation as a result of an increasing accumulation of lipid droplets. Furthermore, lipid droplets were 2.5–8.3 times as stiff as the cytoplasm, but less stiff than the nucleus. Although this is the only published study to address the stiffness of the lipid droplet in comparison to the nucleus, it does so only in adipocytes with relatively small lipid droplets and not adipocytes with excessively large lipid droplets (such as those that would result from fatty acid treatment) that are more representative of the obese condition. Additionally, due to technical limitations of AFM and assumptions made to utilize the Hertz model, absolute values for lipid droplet, nuclear, and cytoplasmic stiffness were not obtained.

In agreement with the aforementioned study, Bouzid et al., demonstrated that differentiated adipocytes were stiffer than preadipocytes with AFM [92]. Moreover, the inhibition of Rho-associated protein kinase (ROCK) with Y-27632 treatment decreased adipocyte stiffness, while Rho activation with CN01 treatment increased stiffness, likely due to the disruption or induction of stress fiber formation, respectively. Interestingly, as determined by traction force microscopy, adipocytes exhibited lower traction forces than undifferentiated preadipocytes, and traction force was further decreased with ROCK inhibition, but remained unchanged with Rho activation. In accordance with observations made previously in hepatocytes [56], this result suggests that the accumulation of lipid droplets disrupts the mechanosensing machinery of the cell, which includes actin stress fibers and focal adhesions.

Interestingly, not all studies have shown that adipocytes are stiffer than their undifferentiated counterparts. Abuhattam et al., demonstrated that Young’s modulus of adipocytes, when subjected to AFM indentation, tended to decrease with time of differentiation, and this was associated with F-actin inhibition [91]. Additionally, Young’s modulus of differentiated adipocytes decreased with increasing lipid droplet size. Using AFM microrheology, the loss tangent (the ratio of loss modulus G”/storage modulus G’) of adipocytes increased at low-frequency oscillations (≤10 Hz) compared to undifferentiated controls, indicating that differentiated cells exhibit more fluid-like behavior. It should be noted, however, that no statistical analysis was performed. The authors also investigated the effect of fatty acid treatment post differentiation on adipocyte stiffness. Young’s modulus decreased when cells were treated with oleic acid, yet it is important to note that cells were fed the fatty acid four days post induction and not after full differentiation was achieved, which typically occurs by day 7–10. More importantly, indentation depth of the AFM tip was less than two microns, and therefore, the AFM studies likely only probed the cell cortex and not whole cell stiffness. On the tissue level, fat bodies of drosophila and gonadal adipose tissue of mice fed high-fat diets were found to be softer than wild-type controls as measured by AFM, in accordance with cell culture experiments. Interestingly, in stark contrast, Young’s modulus of adipose tissue in diabetic mice was greater than wild-type controls, as confirmed by AFM.

Recent evidence suggests that fat accumulation has mechanosensitive consequences on cell function, possibly caused by deformation of the nucleus by the lipid droplet [56]. Future studies should focus on the response of the adipocyte nucleus to mechanical loading. The nucleus alters its shape in response to loading, shrinking under compression and stretching under tension [93]. Nuclear deformation may lead to altered gene expression and cell function. Currently, no computational models predict the extent of nuclear deformation that can occur during adipocyte hypertrophy.

Taken together, these studies demonstrate that test methods—including the instrument of choice, condition of the cell, testing frequency, amongst other parameters—greatly impact the apparent mechanical properties of the adipocyte. However, most studies agree that excess fat accumulation tends to stiffen the cell, which may potentially contribute to the phenomena of tissue stiffening seen in obesity. How adipocyte cell stiffness influences the overall mechanical properties of adipose tissue has not been fully elucidated, but the importance of computational models is emphasized (Table 1).

## 5. Tissue Stiffness Measurements

Just as tissue stiffness is often altered in pathological conditions such as tumorigenesis or fibrosis [11], so is adipose during obesity (Table 2). Adipose tissue from obese mice is at least two-fold stiffer than that from wild-type subjects, as determined by rheometry, and this fold change increases even more when the tissue is compressed [94]. This mechanical phenomenon has been confirmed by an independent study with AFM, which was used to probe the mechanical properties of obesity-associated human diabetic adipose tissue in comparison to non-diabetic adipose [95]. Diabetic adipose tissue was twice as stiff and more variable than the control. Diabetic adipose also demonstrated an increase in collagen content as measured by hydroxyproline, which likely contributed to the increased mechanical properties that was observed. Increased ECM deposition alongside adipocyte hypertrophy is thought to constrain cell size, consequently limiting lipid storage within the adipocyte. As a result, fatty acids are redistributed to vital organs like the liver and heart, contributing to obese-related comorbidities [96]. Interestingly, knockout of collagen VI from obese mice results in improved metabolic function compared to collagen VI-expressing obese controls, despite the adipocytes having unhindered cell growth. This suggests that it is indeed fibrosis that exerts detrimental effects on whole-body homeostasis, and not the accumulation of excess triglycerides in the adipocyte [82].

Matrix stiffness also influences estrogen production. Ghosh et al., demonstrated by seeding adipose-derived stromal cells onto collagen-coated polyacrylamide gels of varying stiffness that matrix compliance leads to an increased production of aromatase, the enzyme responsible for converting androgens into estrogens [97]. This process was facilitated by discoidin domain receptor 1, c-Jun N-terminal kinase, and the proto-oncogene JunB. Moreover, inhibiting cell contractility via pharmaceutical reagents was found to increase aromatase activity and subsequently increase estrogen production. These findings have implications for breast cancer, which is linked to elevated estrogen levels and obesity. The data seem to contradict the prevailing notion that obesity is accompanied by adipose tissue stiffening, not softening. Further investigation is necessary to elucidate the interplay between estrogen production, adipose tissue mechanical properties, and breast cancer.

Not all studies agree that adipose tissue stiffens with obesity. Abuhattum et al., employed AFM to perform indentation and microrheology studies on human preadipocytes during and after complete differentiation [91]. Surprisingly, as the lipid droplet increased in size, the authors found that adipocytes became more compliant. Furthermore, this result was confirmed on the tissue level; fat bodies and gonadal adipose isolated from drosophila and mice, respectively, that were fed high-fat diets were softer than tissue from lean controls, as measured by AFM. Interestingly, in contrast, adipose from a diabetic mouse model (db/db) was stiffer than tissue from lean mice, corroborating the observations made by Wenderott et al. [95]. However, it should be noted that the indentation depth of the AFM was less than two microns, and therefore, the stiffness of the cell cortex was measured and not whole-cell stiffness. Nonetheless, the inconsistent conclusions from these few studies illustrate the fact that tissue stiffness can vary between adipose pathologies, mechanical testing techniques, and testing methods (such as indentation depth, frequency, and rate), amongst many other variables.

Tissue stiffness can also be measured using radiological techniques, such as magnetic resonance or ultrasound elastography, but the paucity of studies on adipose are not discussed here because the methods, frequencies, and measured modulus values are so vastly different from traditional in vitro mechanical testing methods.

## 6. Computational Models of Adipose

Finite element analysis (FEA) is a powerful computational method used to approximate the behavior of physical systems or structures using numerical analysis techniques [98]. Widely used in engineering, physics, and other fields to study the behavior of complex systems, its application has contributed to our understanding of the mechanobiology of adipocytes. The basic principal behind FEA starts with the decomposition of the system of interest into many sub-units or “elements” of simple geometry. Elements are then assigned relevant properties, and all element interactions are mapped with nodal points between them. Boundary and load conditions are applied to represent testing conditions. Multiple types of simulations are run, and one typically mimics a test performed empirically to verify the accuracy of the model. A solution to the model is determined by solving a set of governing equations to obtain the properties of each element and then combining them to determine the system’s overall behavior. Models representing different levels in the hierarchical structure of adipose tissue, from single-cell to whole-tissue analysis, have been developed to further our understanding of adipose mechanics and disease.

### 6.1. Single-Cell Mechanical Models

Single-cell adipocyte models are often used to validate data from in vitro testing and range in complexity depending on the research goal. Material properties for simulation are either determined through in vitro testing or assumptions are made and then applied to the corresponding section of the model. Most current adipocyte research assumes the intracellular components to be homogenous, isotropic, and compressible [90,99]. The subcomponents (plasma membrane, cytoplasm, lipid droplet, nucleus, and substrate) are usually modeled as linearly elastic, but this is an assumption. Although idealized models do not capture exact geometries, they do pull data from empirical testing and the literature to be reasonably representative. However, it must be noted that the amount of experimental data collected on the mechanical properties of cellular components is small, which is a limitation of cellular-level models of mechanical behavior.

Adipocyte geometry and structure have been idealized to improve computational efficiency and enable parameterization of the model. Parameterization allows for the model to vary in critical dimensions across a defined domain, providing a spectrum of results from multiple versions of the same model. Other studies have empirically determined that adipocytes and nuclei are viscoelastic, and therefore, their mechanical behavior is dependent on time and the strain rate [93]. Additionally, numerical convergence may be more difficult to achieve with models that are overly complex.

Shoham et al., examined the mechanical properties of adipocytes filled with lipid droplets at various stages of differentiation and maturation utilizing AFM, interferometric phase microscopy, and 3D finite element models (FEMs) [90]. Variability in cell shape, lipid droplet size and quantity, as well as stage of adipogenesis, was represented by using three different geometries, measured from previously published experimental data. Intracellular components were modeled as truncated spheres and ellipsoids. Loading conditions for these simulations replicated those of the AFM experiments that were presented in the same paper. Overall, the FEMs were able to reproduce the mechanical behavior of adipocytes captured by AFM, and altogether, the results showed that cell stiffness increased with adipogenesis and was dependent on where the cell was probed with the AFM tip (above or adjacent to a lipid droplet or the nucleus). Furthermore, this work demonstrated that lipid droplets are 2.5 to 8.3 times stiffer than the cytoplasm, but softer than the nucleus (lipid droplet stiffness is 0.83 ± 0.14 times nuclear stiffness).

Another study from the same group utilized 3D FEMs of single-cell adipocytes to study the effect of adipocyte differentiation and maturation on large deformation compressive and tensile mechanical behavior. Loading conditions sought to replicate physiologically relevant in vivo mechanics for the cells. The simulations demonstrated that when cells were stretched, intracellular effective Lagrange strains were greater in the cytoplasm than the lipid droplets. With compression simulations, intracellular Lagrange stresses were greater in lipid droplets than the cytoplasm and increased with increasing deformation. Stresses also increased with adipocyte maturity, likely due to a greater intracellular volume of lipid. Moreover, compressive stress in the plasma membrane was larger than in the lipid droplet and cytoplasm, which may hint to a possible mechanism where tensile strains increase plasma membrane permeability, thereby facilitating the cellular uptake of biomolecules involved in mechanotransduction pathways such as MEK (Figure 2), and ultimately influencing adipocyte differentiation [99].

### 6.2. Multi-Cellular Mechanical Models

Simulating single-cell adipocyte mechanics is useful in determining the mechanical behavior of the intracellular components as well as the whole-cell response to mechanical stimuli. A key drawback, however, is that individual, single-cell adipocytes are only relevant to in vitro experiments and do not replicate the same mechanical conditions that exist in vivo. Hence, multi-cellular models of adipose tissue subjected to physiologically relevant loading are more representative of the in vivo condition. Additionally, the mechanical properties of cells are not constant; they continuously and actively remodel their internal structures [100]. To most accurately model adipocyte mechanical behavior, the adaptive response of the adipocyte to its environment must be considered. In addition to stimuli from cell–cell and cell–matrix interactions, adipocytes are indeed mechanosensitive and respond to mechanical stimuli, but a greater understanding of adipocyte mechanosensing, mechanotransduction, and resulting cell response is necessary to build more accurate models.

A model developed by Comley et al., aimed to study the relationship between the microstructure and the macroscopic modulus of porcine subcutaneous adipose tissue. To understand how adipose tissue microstructure changes under load, tissue samples were subjected to 6% tensile or 15% compressive strain, held in position, and then imaged using confocal microscopy [101]. In comparison to unloaded adipose, it was noted that the foam-like structure of adipose was uncompromised, and no rotation or rearrangement of cells was observed. Additionally, it was determined from rheological studies that lipid isolated from adipose tissue is linearly viscoelastic. These data were then used to develop a micromechanical model where adipose tissue was represented as a lipid-filled closed cell foam of reinforced basement membrane and an open cell foam of collagen septa. The model incorporated the moduli of the microstructural units—the septa and basement membrane—to predict tissue stiffness under compression and tension and to understand the role of buckling the cell walls.

Other noteworthy adipose tissue models by Frank et al., evaluated how externally loaded adipocytes impart localized loads onto neighboring cells, which has been hypothesized to stimulate lipid production via the MEK/extracellular signal-regulated kinase (ERK) pathway, promoting hypertrophy of the cell (Figure 3) [102]. Additionally, the effect of adipocyte maturation on localized loads was studied. Two-dimensional FEMs of adipose tissue with a varied adipocyte number (single or multiple) at different differentiation stages were constructed to simulate the external compression of the cells and to determine strain energy density at the plasma membrane. The simulations demonstrated that when the tissue was externally loaded, a given adipocyte was deformed by its neighbors and the extent of deformation increased with adipocyte differentiation.

Although tissue-level models are intended to recapitulate in vivo mechanical behavior, they are validated using empirical data from in vitro testing. More complex than single-cell models, tissue models depend on assumptions and idealizations to save computational cost. Yet, modeling of both cellular-level and tissue-level behavior is necessary to understand the complexity of adipocytes and adipose tissue. As mechanosensing, mechanotransduction, and the active response of adipocytes are more commonly studied and better understood, computational models will likely evolve to have better predictive power.

## 7. Conclusions

Adipocytes are mechanosensitive. They generate and respond to forces in their surrounding environment, thereby affecting cellular processes such as differentiation, insulin transport, and lipid accumulation. Although it has been demonstrated in many other cell types that changes in the mechanical environment of a cell can initiate and progress disease, the role of adipocyte mechanobiology in obesity has not yet been well established. However, a growing body of research shows that adipocytes express mechanosensitive proteins such as YAP/TAZ, piezo1/2, and SWELL1, that they respond to changes in underlying substrate stiffness or external loading, and that their cell stiffness increases with maturation and increasing lipid droplet size. Although there are few cellular- or tissue-level models, there is growing interest in simulating the mechanical behavior of adipocytes and adipose under applied loads.

Obesity has been predominantly viewed as an inflammatory condition, but has been recently acknowledged as having mechanobiological underpinnings as discussed in this review, albeit the number of published studies is limited. Future research should aim to deepen our understanding of how adipocytes sense mechanical cues and the signaling pathways involved. Furthermore, quantification of in vivo loads and deformations alongside the development of more accurate computational models would help better simulate and forecast adipocyte and adipose tissue mechanical behavior. Through this approach, we can uncover mechanobiological mechanisms associated with normal function and disease, potentially identifying therapeutic targets and interventions for obesity treatment and management.

## Figures and Tables

**Table 1:** Summary of methods and results from peer-reviewed publications within the last five years that measure adipocyte cell stiffness.

Publication	General Protocol	Samples	Cell Stiffness
Abuhattam, S., Taubenberger, A.V. et al., Scientific Reports 2022 [67]	**AFM** 5 μm spherical tip 5 μm/s indentation rate to 2.5 nN Hertz/Sneddon model	Day 1 of differentiation, single-cell SGBS adipocytes (control)	E = 3.84 ± 0.15 kPa
		Day 11 of differentiation, single-cell SGBS adipocytes	E = 0.46 ± 0.02 kPa
	**AFM Microrheology** 5 μm spherical tip 2 nN initial setpoint, 10 nm amplitude oscillations at 3–200 Hz frequency	Day 1 of differentiation, single-cell SGBS adipocytes (control)	G’ = ~1–1.25 kPa G” = ~0.125–1.25 kPa
		Day 1 of differentiation, single-cell SGBS adipocytes	G’ = ~0.125–0.5 kPa G” = ~0.075–0.13 kPa
Bouzid, T., Lim, J.Y. et al., Biochemical and Biophysical Research Communications 2022 [68]	**AFM** Triangular pyramid tip Indented to 5 nN Hertz Pyramid model	3T3-L1 preadipocytes (control)	E = ~4 kPa (probing above nucleus or cytoplasm)
		Differentiated 3T3-L1 adipocytes	E = ~8 kPa (probing above nucleus or lipid droplet)

AFM = atomic force microscopy, SGBS = Simpson–Golabi–Behmel Syndrome, E = Young’s modulus, G’ = storage modulus, G” = loss modulus.

**Table 2:** Summary of methods and results from peer-reviewed publications within the last five years that measure adipose tissue stiffness.

Publication	General Protocol	Samples	Tissue Stiffness
Abuhattam, S., Taubenberger, A.V. et al., Scientific Reports 2022 [67]	**AFM** 10 μm spherical tip 5 μm/s indentation rate to 2.5 nN Hertz/Sneddon model	Gonadal WAT from 46 wk old male C57BL/6-J mice (control)	E = ~2 kPa
		Gonadal WAT from HFD-fed, 46 wk old male C57BL/6-J mice	E = ~0.9 kPa
		Gonadal WAT from 18 wk old male C57BL/6-J mice (control)	E = ~0.5 kPa
		Gonadal WAT from 18 wk old male db/db mice	E = ~1.2 kPa
		Gonadal WAT from 18 wk old male ob/ob mice	E = ~0.5 kPa
	**AFM** 10 μm spherical tip 5 μm/s indentation rate to 4 nN Hertz/Sneddon model	Fat bodies from WT Drosophila (control)	E = ~0.4 kPa
		Fat bodies from HFD-fed Drosophila	E = ~0.25 kPa
	**AFM Microrheology** 10 μm spherical tip 4 nN initial setpoint, 10 nm amplitude oscillations at 5–60 Hz frequency	Gonadal WAT from 46 wk old male C57BL/6-J mice (control)	G’ = ~0.4–0.6 kPa G” = ~0.05 kPa
		Gonadal WAT from HFD-fed, 46 wk old male C57BL/6-J mice	G’ = ~0.2–0.4 kPa G” = ~0.01–0.12 kPa
		Gonadal WAT from 18 wk old male C57BL/6-J mice (control)	G’ = ~0.5 kPa G” = ~0.05–0.2 kPa
		Gonadal WAT from 18 wk old male db/db mice	G’ = ~0.5–0.85 kPa G” = ~0.05–0.35 kPa
	**Magnetic resonance elastography** 400–1400 Hz drive frequencies 2000 ms repetition time, 42 ms echo time, 3 mm slice thickness, 64 × 64 matrix size, (9.6 × 9.6) mm^2^ field of view, (0.15 × 0.15 × 3) mm^3^ voxel size Bessel function	Gonadal WAT from 46 wk old male C57BL/6-J mice (control)	G’ = ~1–1.5 kPa G” = ~1–2 kPa
		Gonadal WAT from HFD-fed, 46 wk old male C57BL/6-J mice	G’ = ~0.9–1.5 kPa G” = ~0.9–2.1 kPa
		Gonadal WAT from 18 wk old male C57BL/6-J mice (control)	G’ = ~1 kPa G” = ~0.5–3 kPa
		Gonadal WAT from 18 wk old male db/db mice	G’ = ~1–2 kPa G” = ~1–2.5 kPa
Wenderott, J.K, O’Rourke, R.W. et al., Scientific Reports 2020 [71]	**AFM** Triangular pyramid tip Hertz model	VAT from non-diabetic human females (control)	E = 4.48 ± 4.81 kPa
		VAT from diabetic human females	E = 11.50 ± 16.79 kPa

AFM = atomic force microscopy, WAT = white adipose tissue, WT = wild-type, HFD = high-fat diet, VAT = visceral adipose tissue, E = Young’s modulus, G’ = storage modulus, G” = loss modulus.

## Data Availability

Not applicable.
